# Axonal Transport of Lysosomes Is Unaffected in Glucocerebrosidase-Inhibited iPSC-Derived Forebrain Neurons

**DOI:** 10.1523/ENEURO.0079-23.2023

**Published:** 2023-10-06

**Authors:** A. J. Keefe, D. R. Gabrych, Y. Zhu, D. J. Vocadlo, M. A. Silverman

**Affiliations:** 1 Department of Biological Sciences, Simon Fraser University, Burnaby, British Columbia V5A 1S6, Canada; 2 Centre for Cell Biology, Development, and Disease, Simon Fraser University, Burnaby, British Columbia V5A 1S6, Canada; 3 Department of Chemistry, Simon Fraser University, Burnaby, British Columbia V5A 1S6, Canada; 4 Department of Molecular Biology and Biochemistry, Simon Fraser University, Burnaby, British Columbia V5A 1S6, Canada

**Keywords:** axonal transport, conduritol B epoxide, glucocerebrosidase, lysosomes

## Abstract

Lysosomes are acidic organelles that traffic throughout neurons delivering catabolic enzymes to distal regions of the cell and maintaining degradative demands. Loss of function mutations in the gene *GBA* encoding the lysosomal enzyme glucocerebrosidase (GCase) cause the lysosomal storage disorder Gaucher’s disease (GD) and are the most common genetic risk factor for synucleinopathies like Parkinson’s disease (PD) and dementia with Lewy bodies (DLB). GCase degrades the membrane lipid glucosylceramide (GlcCer) and mutations in *GBA*, or inhibiting its activity, results in the accumulation of GlcCer and disturbs the composition of the lysosomal membrane. The lysosomal membrane serves as the platform to which intracellular trafficking complexes are recruited and activated. Here, we investigated whether lysosomal trafficking in axons was altered by inhibition of GCase with the pharmacological agent Conduritol B Epoxide (CBE). Using live cell imaging in human male induced pluripotent human stem cell (iPSC)-derived forebrain neurons, we demonstrated that lysosomal transport was similar in both control and CBE-treated neurons. Furthermore, we tested whether lysosomal rupture, a process implicated in various neurodegenerative disorders, was affected by inhibition of GCase. Using L-leucyl-L-leucine methyl ester (LLoME) to induce lysosomal membrane damage and immunocytochemical staining for markers of lysosomal rupture, we found no difference in susceptibility to rupture between control and CBE-treated neurons. These results suggest the loss of GCase activity does not contribute to neurodegenerative disease by disrupting either lysosomal transport or rupture.

## Significance Statement

Lysosomal trafficking is an integral process for the proper functioning and maintenance of neuronal axons and synapses. Loss of function mutations in the lysosomal enzyme glucocerebrosidase (GCase) are implicated in a number of neurodegenerative disorders. The mechanisms underlying how impairment of GCase activity results in these pathogenic processes are not well understood. Here, we show that two processes commonly implicated in various lysosome-related disorders, lysosomal transport and rupture, are unaffected in human induced pluripotent human stem cell (iPSC)-derived forebrain neurons treated with Conduritol B Epoxide (CBE), a pharmacological inhibitor of GCase. Thus, GCase dysfunction and the resulting pathogenesis must be related to intraneuronal processes other than lysosomal transport and rupture.

## Introduction

Lysosomes are acidic vesicles specializing in the degradation of damaged cellular material, such as oxidized macromolecules and defective organelles. The acidic interior of lysosomes carries catabolic enzymes that break down biological polymers, such as proteins, lipids, and carbohydrates, into their monomeric components. In the axon of neurons, lysosomes and other organelles such as late endosomes or autophagosomes (collectively referred to as lysosomes for simplicity), survey synapses in search of damaged material, which they collect and prepare for degradation. Lysosomal degradation of cargo initiates in synapses, but degradative efficiency increases as they transport toward the soma, where lysosomes are more acidic and numerous providing greater degradative capacity (Lie et al., 2021). Within the narrow confines of the axon, constitutive disposal of damaged materials is a priority because they otherwise block transport by accumulating into large deposits that manifest as axonal varicosities; a known pathophysiological marker seen in multiple neurologic disorders (Lee et al., 2011; Gowrishankar et al., 2015). Dysfunction in the trafficking of lysosomal cargo essential to fulfill degradative demands contributes to the pathogenesis of major neurodegenerative disorders such as Gaucher’s disease (GD) and Parkinson’s disease (PD; Wong and Krainc, 2016). Lysosomal membrane composition, which is altered in GD and other lysosomal disorders (Hein et al., 2008, 2017; Fuller and Futerman, 2018), influences lysosomal trafficking by regulating the recruitment of motor proteins and their adaptors (Appelqvist et al., 2011; Wong and Krainc, 2016). For example, membrane cholesterol influences vesicle transport by tuning the inhibitory effect of the microtubule-associated protein tau on kinesin cargo binding (Q. Li et al., 2023). Lipid dyshomeostasis can drive dysfunction in motor protein recruitment or perturbations in lysosomal membrane integrity. Impairment of either of these processes could compromise lysosomal axonal transport to cause a decline in axonal and synaptic proteostasis, thereby contributing to the pathogenesis of various neurodegenerative diseases.

Glucocerebrosidase (GCase) is a lysosomal enzyme encoded by the *GBA* gene. The principal function of GCase is to catalyze the degradation of the glycosphingolipid (GSL) glucosylceramide (GlcCer) into glucose and ceramide. GSLs are glycolipids that are abundant within the brain and found within the cellular membrane. Homozygous loss of function mutations in *GBA* cause the lysosomal storage disorder GD (Montfort et al., 2004; Hruska et al., 2008), but carrying a single mutant allele is also a major risk factor for a group of neurodegenerative disorders termed synucleinopathies (Lwin et al., 2004; Alcalay et al., 2014; Shiner et al., 2021), which includes PD and dementia with Lewy bodies (DLB). The mechanisms by which *GBA* mutations cause or promote neurodegenerative disease remain poorly understood but may be related to the lysosomal accumulation of its substrate GlcCer, and its deacetylated derivative glucosylsphingosine (GlcSph; Lunghi et al., 2022; Navarro-Romero et al., 2022). Alternative pathogenic mechanisms of *GBA* mutations are reviewed elsewhere (Gegg et al., 2022). The membranous accumulation of GlcCer and GlcSph may disrupt axonal transport dynamics, for example, by interfering with motor-membrane interactions or increasing the permeability of lysosomes and promoting rupture. Such a potential effect is precedented in Niemann Pick type C, where accumulation of cholesterol within the lysosomal membrane impairs axonal trafficking of these organelles (Roney et al., 2021). Thus, mutations or catalytic inhibition of GCase may contribute to neural dysfunction by interfering with lysosomal trafficking, a hypothesis that has not yet been examined. Conduritol B Epoxide (CBE) is a pharmacological agent that acts by selectively inactivating GCase by forming a covalent adduct with the catalytic residues of this enzyme, leading to a complete loss of function (Shou-jun et al., 1985). Accordingly, CBE is a useful tool to study the link between GCase in both GD and PD (Mus et al., 2019; Henderson et al., 2020). Notably, repeated administration of CBE to mice phenocopies GD (Vardi et al., 2016) and has been used to explore the link between GCase and PD (Mus et al., 2019; Henderson et al., 2020) showing in several cases concordance between genetic malfunction of GCase and its pharmacological antagonism (Farfel-Becker et al., 2019).

In this study, we show that pharmacological inhibition of GCase using CBE in induced pluripotent human stem cell (iPSC)-derived forebrain neurons has no effect on the transport of lysosomes throughout the axon. Thus, we hypothesized that other factors related to lysosomal membrane composition, such as lysosomal rupture, may be influenced by inhibition of GCase. We performed biochemical assays to assess whether pharmacological inhibition of GCase in neural progenitor cells (NPC), or iPSC-derived forebrain neurons affected this parameter. We found that lysosomal rupture was unaffected. Further studies will be required to precisely define the pathways by which inhibition or dysfunction of GCase contributes mechanistically to lysosomal-related neuronal dysfunction.

## Materials and Methods

### Differentiation and cell culture of iPSC-derived neurons and expression of transgenes

iPSCs (gift of F. Lynn, University of British Columbia) from a healthy male donor were plated onto Matrigel (Corning, 354277) coated plates and cultured in mTeSR Plus (STEMCELL Technologies, 100-0276). iPSCs were differentiated into NPCs according to protocols from STEMCELL Technologies. Briefly, iPSCs were passaged into STEMdiff Neural Induction Media + SMADi (STEMCELL Technologies, 08581) and 10 μm ROCK inhibitor Y-27 632 (STEMCELL Technologies, 72302) for 6 d with daily media exchanges. Neural Progenitor Cells were plated onto Matrigel coated plates and cultured in NPC Complete Media (STEMCELL Technologies, 05833) for up to eight passages. For differentiation into neurons, NPCs were passaged onto Poly-L-Ornithine (PLO; Sigma, A-004-C) and Laminin (Sigma, L2020) coated plates in NPC Forebrain Differentiation Media (STEMCELL Technologies, 08600) for 5 d. Differentiated NPCs were passaged onto PLO/Laminin coated coverslips in 12-well plates in Forebrain Neuron Maturation Media (STEMCELL Technologies, 08,605). During maturation, neurons received half media exchanges every 3 d and were used for experiments on day 30 of maturation. For each independent experiment, a new vial of NPCs was thawed and used for differentiation into terminally differentiated neurons.

Transfections were conducted on neurons at 54 d *in vitro* (DIV) using EndoFectin (GeneCopoeia, EF013). For each coverslip, 0.5 μg of DNA was added to 60 μl of OptiMEM (ThermoFisher, 31985062) + 2.5 μl of EndoFectin and incubated for 20 min. Cell medium was replaced with 500-μl OptiMEM and the transfection mixture was added dropwise to each well. After a 1-h incubation, the transfection mixture was removed and reserved media returned. Transgenes were allowed 24–48 h to express before live-cell imaging.

### CBE and GCase activity assay

The GCase inhibitor Conduritol B Epoxide (CBE; Cayman Chemicals, 6090-95-5) was reconstituted at 3 mm in dH_2_O and stored at −80°C until use. On day 20 of neuronal maturation, the medium was supplemented with 100 μm CBE that was replenished every 3 d with media exchanges. A CBE dose of 100 μm was used because it is sufficient for complete GCase inhibition and causes GlcCer accumulation (Korkotian et al., 1999; Henderson et al., 2020). After 10 d of CBE treatment, neurons were washed with PBS and treated with the GCase substrate LysoFQ-GBA (Deen et al., 2022) at 5 μm for 1 h. The neurons were washed two times for 3 min each in PBS containing the GCase inhibitor AT3375 (Deen et al., 2022) and mounted into live-cell imaging chambers for fluorescence microscopy.

### Live cell imaging and fluorescence microscopy

For live-cell imaging, iPSC-derived neurons were mounted in a heated chamber and imaged using a wide-field fluorescence microscope (DMI, 6000B Leica) equipped with a CCD camera (Hamamatsu Orca-ER-1394). Neurons were imaged in prewarmed BrainPhys Imaging Optimized Media (STEMCELL Technologies, 05790) with a 63×/1.40 lens using immersion oil (Cargille; type DF, 16242). As previously performed (Gan et al., 2020), axons were initially identified based on morphology and imaged ∼150–200 μm from the cell body. Subsequently, a sample of cells were immunostained after live imaging to retrospectively confirm axon identity either by colocalization with Tau (1:500, ThermoFisher, AHB0042), an axonal protein, or absence of MAP2 (1:700, Sigma, AB5622), a dendritic cytoskeletal protein. In order to determine the orientation of the cell body relative to the axon, and thus to distinguish between anterograde and retrograde transport events, only visually isolated axons were imaged. Movies were acquired in MetaMorph (Molecular Devices). For LAMP1-eGFP trafficking dynamics [gift of G. Banker (retired), Oregon Health and Sciences University], frames were acquired at 0.75-s intervals (1.25 frames/s) for 120 s. For MTS-dsRed2 (gift of G. Rintoul, Simon Fraser University) trafficking dynamics, frames were acquired at 2-s intervals (0.5 frames/s) for 3 min. For LysoBrite imaging, neurons were treated for 5 min in 1:5000 solution of LysoBrite (Cayman Chemical, 25157) washed two times for 2 min each in imaging media, and imaged at 1-s intervals (one frame per second) for one and a half minutes. Video analyses are described below.

For immunocytochemistry, images were acquired as 3-μm Z-stacks with 0.5-μm intervals using either 40× or 63× lens. For GFAP, LysoBrite, and LAMP1 intensity analyses, random fields of view were acquired using the same imaging parameters between coverslips.

### LLoME assay

The lysosomotropic detergent L-Leucyl-L-Leucine methyl ester (LLoME; Cayman Chemical, 16008) was freshly prepared in PBS before use. NPCs were incubated in 600 μm LLoME in OptiMEM, or OptiMEM without LLoME, for 1 h. After, cells were washed twice with PBS and incubated for 1 h in NPC complete media and then fixed in 4% paraformaldehyde (PFA) + 4% sucrose for 15 min at 37°C. For LLoME recovery assays, NPCs were fixed after 24 h.

### Immunocytochemistry

Cells were fixed in 4% PFA + 4% sucrose for 15 min at 37°C. Cells were permeabilized in 0.1% triton in PBS for 1 h. For LAMP1, 0.1% saponin was added to all subsequent staining steps. Cells were blocked for 1 h in 5% BSA/0.5% fish-skin gelatin. Primary antibodies were diluted in blocking solution at the following concentrations: mouse α-Nestin (1:1000, STEMCELL Technologies, 60091), rabbit α-PAX6 (1:500, Sigma, 030775), mouse α-SOX2 (1:200, Developmental Studies Hybridoma Bank, PCRP-SOX2-1B3), mouse α-LAMP1 (1:100, Developmental Studies Hybridoma Bank, H4A3-s), rabbit α-GFAP (1:500, ThermoFisher, PA1-10019), rabbit α-P62 (1:300, Sigma, P0067), rabbit α-LC3B (1:300, Cell Signaling, 3868), rabbit α-MAP2 (1:700, Sigma, AB5622), mouse α-PHF Tau (1:700, ThermoFisher, MN1020), guinea pig α-SV2A (1:500, Synaptic Systems, 119004), and chicken α-Synapsin 1/2 (1:800, Synaptic Systems, 106006). Cells were incubated in primary antibodies at 4°C overnight and then washed three times for 5 min each in PBS. Cells were incubated in the species-appropriate secondary antibodies for 1 h at room temperature: α-rabbit Cy5 (1:800, Jackson ImmunoResearch, 711-175-152), α-chicken Alexa 488 (1:800, Synaptic Systems, N0702-At488-S), α-rabbit Cy3 (1:800, Jackson ImmunoResearch, 111-165-144), or α-Guinea Pig Cy5 (1:800, Jackson ImmunoResearch, 706-175-148). Cells were washed for 10 min in 0.5 μg/ml 4′,6-diamidino-2-phenylindole (DAPI) in PBS for visualization of nuclei, and then washed three times for 5 min each in PBS. Coverslips were mounted onto glass slides using elvanol mounting medium and imaged using widefield fluorescence microscopy.

### Image analysis

Trafficking videos were converted into kymographs and analyzed using custom software (Kwinter and Silverman, 2009). Trafficking parameters, including flux, velocity, and run length, were quantified for anterograde and retrograde events by manually tracing particle movements on kymographs. To determine the percentage of stationary vesicles that moved <5 μm over the course of the 90-s videos, vesicles were manually counted. To analyze lysosomal density, all movement events and stationary lysosomes were summed and divided by the total length of the kymograph for each video. Flux was defined as the total distance traveled by a vesicle standardized by the length and duration of each movie (in microminutes): $\frac{\sum_{i=1}^{n}d_{i}}{l\times t}$ where d are the individual vesicle run lengths, l is the length of axon imaged and t is the duration of the imaging session. A lysosomal punctum was defined as undergoing a directed run if it traveled a distance of ≥5 μm. This distance was determined as a safe estimate of the limit of diffusion based on the assumption that root-mean-squared displacement equals $\sqrt{2D}t$, where D is the diffusion coefficient (D = 0.01 μm^2^/s) and t is the duration of the imaging period (*t* = 50 s; Abney et al., 1999; Gan and Silverman, 2016). A run was defined as terminating if the vesicle remained in the same position for at least four consecutive frames. Kymograph distances were calibrated to 630× magnification (1 pixel = 0.160508 μm).

Images from LLoME assays were analyzed in ImageJ using the *ComDet* (v1.1.3) plugin. *ComDet* is an open-source semi-automated puncta colocalization plugin that uses automatic thresholding and segmentation capabilities to measure puncta size, intensity, and colocalization between color channels (https://github.com/ekatrukha/ComDet). Briefly, images from the Texas Red channel (LAMP1) were merged with images from the FITC channel (p62 or LC3) producing a color stack. The ImageJ plugin Stack Focuser (https://imagej.nih.gov/ij/plugins/stack-focuser.html) was used to generate focused Z-projected images. Cells were outlined by hand using the ImageJ “Region of Interest (ROI)” tool, and then analyzed using the ImageJ plugin *ComDet*. *ComDet* analysis parameters (minimum size, colocalization maximum distance, and intensity above background threshold) were identical between all images analyzed. p62 and LC3 puncta that did not colocalize with LAMP1 were considered background or artifacts and removed from the analysis.

Whole 63× field-of-view analysis of GCase substrate signal and GFAP staining intensity was conducted in *MetaMorph*. Z-stacks were projected into single planes using the Best Focus projection algorithm. A threshold of 3× background intensity was applied to images, and average pixel intensity, integrated pixel intensity, and total pixel area were analyzed using Region Statistics in *MetaMorph*. For GCase activity assays, the number of cells per field of view was manually counted from corresponding phase-contrast images.

### Statistical analysis

All data were assembled and organized in *Excel* (Microsoft) before importing into *RStudio* (RStudio) for analysis using the statistical programming language R. Statistical differences between groups were analyzed by an unpaired Student’s *t* test with equal or unequal variances at a 95% confidence interval. All data are presented as mean ± SEM with data points plotted in the background. For lysosomal rupture experiments, we performed one-way ANOVAs with Tukey’s *post hoc* tests, comparing control and CBE-treated conditions within the same experiments. A statistical table for all analyses is provided in Extended Data Table 1-1.

## Results

### CBE inhibits GCase activity but does not affect axonal transport of lysosomes

**Figure 1.**
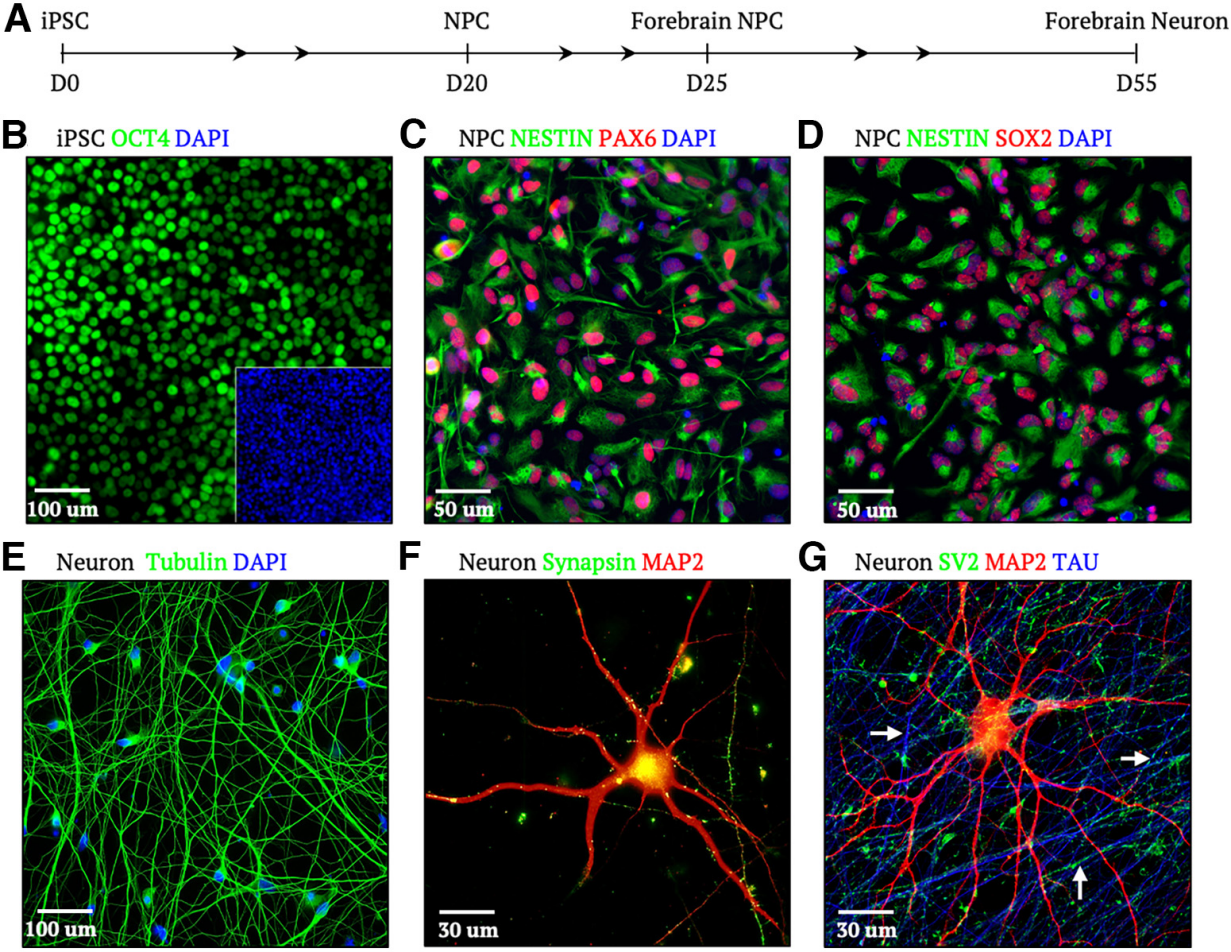
Differentiating and characterizing iPSC-derived neurons. ***A***, Timeline of iPSC culture and differentiation from day 0 (D0) to NPCs by D20, and subsequent differentiation to forebrain NPCs (D25) and forebrain neurons (D55). ***B***, iPSCs express the pluripotency marker OCT4 (green). ***C***, ***D***, NPCs express the neural lineage markers NESTIN (green), PAX6 (red), and SOX2 (red). ***E***, Differentiated neuron cultures stained for tubulin (green). ***F***, Terminally differentiated neurons express the presynaptic protein synapsin (green) and dendritic protein MAP2 (red). ***G***, Terminally differentiated neurons express the axonal protein Tau (blue) and the presynaptic protein SV2 (green).

Using a small molecule-mediated differentiation protocol, iPSCs were differentiated into NPCs and ultimately neurons over a course of 55 d (Fig. 1*A*). iPSC identity was confirmed by immunostaining of the pluripotency marker OCT4. Differentiation of iPSCs to NPCs was confirmed by immunostaining for expression of the markers Nestin, PAX6, and SOX2 (Fig. 1*C*,*D*). Following differentiation and maturation of NPCs to forebrain neurons, we conducted immunocytochemical staining for neuronal identity and polarity markers including the dendritic and axonal markers MAP2 and tau, respectively, as well as the synaptic markers synapsin and SV2. At day 55, neurons showed clear staining for four maturation markers, MAP2, MAPT/tau, synapsin, and SV2, confirming the generation of polarized neurons for subsequent transport studies (Fig. 1*E–G*).

10.1523/ENEURO.0079-23.2023.f1-1 Extended Data Figure 1-1 CBE does not influence LC3 staining following LLoME treatment. CBE-treated NPCs and controls assessed for lysosomal rupture events by staining for LAMP1 (top, red) and LC3 (middle, green), and colocalized (bottom). ***A***, ***B***, In the absence of LLoME, neither CBE nor control NPCs develop LC3-positive lysosomes. ***C***, ***D***, Following treatment of 600 μm LLoME for 1 h, LC3 puncta form in both CBE and control conditions. ***E***, LC3-positive puncta colocalizing with LAMP1 puncta were analyzed using the ImageJ plugin *ComDet*, revealing no significant difference in puncta relative fluorescence units (RFU) per cell. ***F***, The percent of LAMP1 positive puncta that colocalized with LC3 puncta, indicating lysosomal rupture, per cell were analyzed using *ComDet*. There was no significant difference in the percent ruptured lysosomes per cell between controls and CBE-treated NPCs (*N* = 3 independent experiments run in duplicate; Control +LLoME, *n* = 90 cells; Control −LLoME, *n* = 32 cells; CBE +LLoME, *n* = 68 cells; CBE −LLoME, *n* = 36 cells; Extended Data Table 1-1). Download Figure 1-1, TIF file.

10.1523/ENEURO.0079-23.2023.tab1-1 Extended Data Table 1-1 Student *t* test *p* values for all data. Download Table 1-1, DOC file.

**Figure 2.**
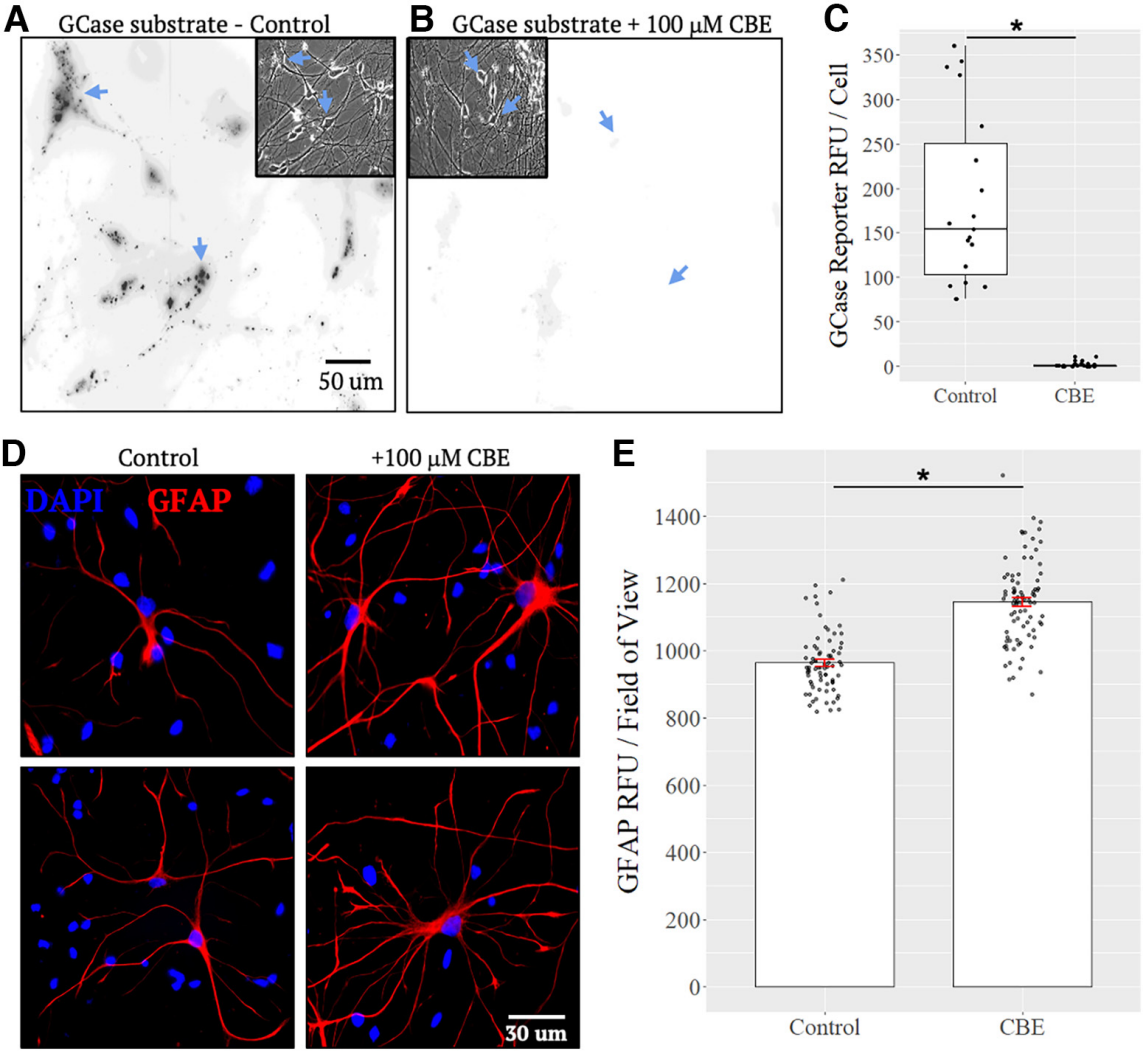
Validation of GCase inhibition with CBE. ***A***, ***B***, Relative to controls, 10-d CBE-treated neurons incubated with the GCase substrate *GBA*1-FQ6 show negligible fluorescence activity. ***C***, Relative fluorescence units (RFU) per cell. *N* = 3 independent experiments run in duplicate; Control, *n* = 19 fields of view; CBE, *n* = 21 fields of view. CBE treatment (100 μm) significantly reduces GCase activity relative to controls (*p < 0.0001; Extended Data Table 1-1). ***D***, Ten days of 100 μm CBE treatment significantly upregulates the staining intensity of astrocytic marker GFAP compared with controls. ***E***, Average GFAP staining intensity per field of view. *N* = 3 independent experiments run in duplicate; Control, *n* = 112 fields of view; CBE, *n* = 120 fields of view. Average GFAP staining intensity per field of view is significantly increased in CBE-treated cultures (*p < 0.0001; Extended Data Table 1-1).

CBE is a well-characterized irreversible inhibitor of GCase that is frequently used to pharmacologically model acute GCase deficiency *in vitro* and *in vivo* (Prence et al., 1996; Farfel-Becker et al., 2019). Before application in downstream transport studies, we confirmed efficacy of CBE in eliminating GCase activity within neurons. Neurons were incubated with 100 μm CBE, and on day 10, cultures were treated with LysoFQ-GBA (Deen et al., 2022), a selective fluorescence-quenched substrate of GCase that can be used to quantify its activity within lysosomes of live cells, and imaged via wide-field fluorescence microscopy (Fig. 2*A*,*B*; Extended Data Table 1-1). A CBE dose of 100 μm was used because it is sufficient for complete GCase inhibition and causes GlcCer accumulation (Korkotian et al., 1999; Henderson et al., 2020). Cultures treated with CBE had significantly reduced LysoFQ-GBA signal compared with control (*p* < 0.001; Fig. 2*C*; Extended Data Table 1-1). Consistent with established differentiation protocols (STEMCELL Technologies), ∼10% of the culture consisted of astrocytes which was confirmed via quantification of GFAP-positive astrocytes in control cultures (data not shown). A common observation in models of GCase deficiency is the activation of GFAP-positive astrocytes, indicated by GFAP upregulation (Niederkofler et al., 2019; Aflaki et al., 2020; Pewzner-Jung et al., 2021). To confirm that CBE affected GCase activity, we assessed the staining intensity of GFAP-positive astrocytes following a 10-d treatment with CBE. We found that GFAP staining intensity was significantly upregulated in CBE-treated cultures (*p* < 0.001; Fig. 2*D*,*E*; Extended Data Table 1-1), consistent with other models of GCase deficiency (Aflaki et al., 2020). The upregulation of GFAP staining intensity, a marker of glial activation, supports CBE having expected pharmacological effects in our cultured cell model.

**Figure 3.**
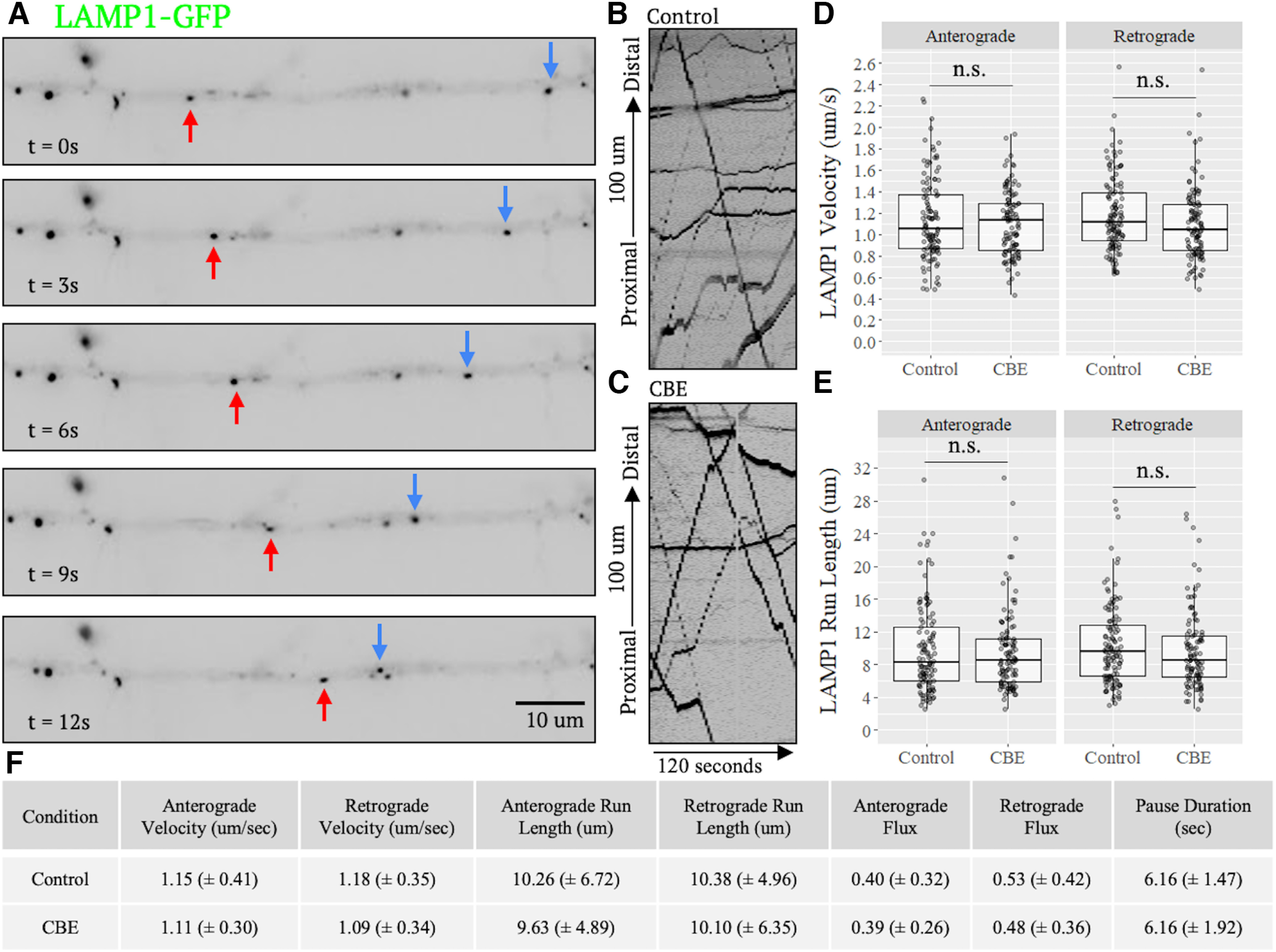
CBE does not affect axonal transport of LAMP1-GFP vesicles. 54 DIV neurons were transfected with the lysosome marker LAMP1-GFP and imaged 24 h later. ***A***, Still frames of a representative axon from a control video sampled at various time points (Movie 1) and (***B***, ***C***) representative kymographs from one control and one CBE-treated LAMP1-GFP video. Red arrows denote retrograde; blue arrows denote anterograde. Videos were acquired at 1.5 frames/s for 120 s. ***D***, ***E***, Analysis of kymographs revealed no significant effect of CBE treatment on transport parameters (*N* = 4 independent experiments run in duplicate; Control, *n* = 116 cells, 2080 transport events; CBE, *n* = 109 cells, 2179 transport events, n.s. = nonsignificant, *p* > 0.05). ***F***, Table of all measured transport parameters (±SD) and Extended Data Table 1-1.

The loss of GCase activity causes accumulation of GlcCer within the membranes of organelles, particularly lysosomes (Prence et al., 1996; Korkotian et al., 1999; Hein et al., 2007). Because the lipid composition of vesicles can affect their transport dynamics (Pathak and Mallik, 2017), we investigated whether axonal transport of lysosomes would be affected by CBE treatment. Following a 10-d treatment with CBE, 54 DIV neurons were transfected with LAMP1-GFP, a marker of lysosomes and predegradative lysosomal populations (late endosomes and autophagosomes), and allowed to express this marker for 24 h before imaging. Movies were captured from axons and converted into kymographs for analysis of vesicle velocity, run length, flux, pause duration, and directionality (Fig. 3*A*,*B*; Extended Data Table 1-1; Movie 1). LAMP1-GFP vesicles were abundant in the axon and trafficked bidirectionally. All transport parameters examined were not statistically significant in either the anterograde or retrograde direction compared with controls (Fig. 3*C–E*; Extended Data Table 1-1). There was no bias in directionality, consistent with previous reports (Klinman and Holzbaur, 2016), and a slight decrease in the velocity, run length, and flux in the retrograde direction in CBE-treated neurons was observed, but was not significant.

Movie 1. Live imaging of axons showing LAMP1-eGFP transport in control versus CBE-treated cells. 1.25 frames/s, 20-s recording, playback: 1× real time. 10.1523/ENEURO.0079-23.2023.video.1

**Figure 4.**
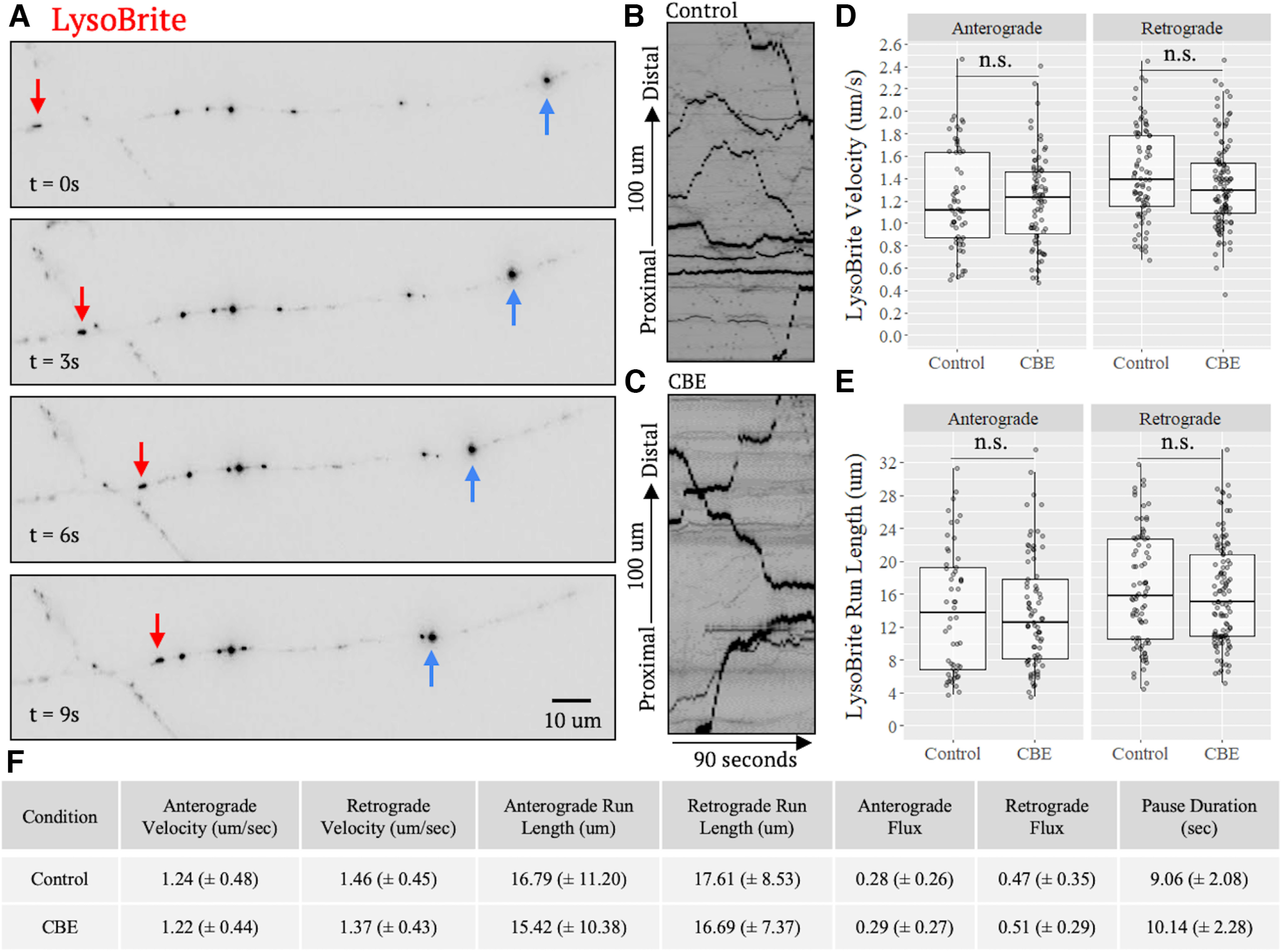
CBE does not affect axonal transport of acidic vesicles. 55 DIV neurons were stained with LysoBrite and imaged live. ***A***, Still frames of one representative axon from a control condition transport video sample at various time points and (***B***, ***C***) representative kymographs from one control and one CBE-treated LysoBrite transport video. Red arrows denote retrograde; blue arrows denote anterograde. Videos were acquired at one frame per second for 90 s. ***D***, ***E***, Analysis of kymographs revealed no significant effect of CBE treatment on any transport parameter (*N* = 3 independent experiments run in triplicate; Control, *n* = 116 cells, 752 transport events; CBE, *n* = 140 cells, 1180 transport events, n.s. = nonsignificant, *p* > 0.05). ***F***, Table of all measured transport parameters (±SD) and Extended Data Table 1-1.

Although LAMP1 primarily localizes to mature lysosomes, it is also expressed in other vesicles including late endosomes or trans-Golgi carrier vesicles (Lie et al., 2021). Overall, ∼20% of LAMP1 vesicles within the axon colocalize with LysoTracker, a marker that primarily stains mature lysosomes (Gowrishankar et al., 2021). As GCase activity depends on an acidic pH, trafficking dysfunction associated with its inhibition may be restricted to mature lysosomes. It was possible that the slight trend toward transport deficits noted above when using LAMP1 as a marker may have been because of a preferential transport deficit in mature lysosomes that was obscured by the breadth of LAMP1 staining. Therefore, to assess the transport of mature lysosomes, we used LysoBrite, a variant of LysoTracker that accumulates within the acidic lumen of mature lysosomes and is better suited for live-cell imaging of transport dynamics because of its enhanced photostability and brightness relative to GFP. Of note, although LysoBrite and other pH sensitive dyes are more specific for lysosomes than LAMP1-GFP, they also may label slightly acidic autophagosomes or late endosomes. Neurons were plated on coverslips and treated with CBE for 10 d. 55 DIV neurons were then stained with LysoBrite for analysis of trafficking (Fig. 4*A*,*B*; Extended Data Table 1-1). Similar to LAMP1, LysoBrite-stained vesicles showed no significant difference in CBE-treated neurons compared with controls in any transport parameters in both anterograde and retrograde directions (Fig. 4*C–E*; Extended Data Table 1-1). A slight, but not significant, bias in retrograde directionality of vesicles was seen. These results collectively show that axonal transport dynamics of mature lysosomes are not significantly affected by CBE treatment.

### CBE does not affect axonal transport of mitochondria

**Figure 5.**
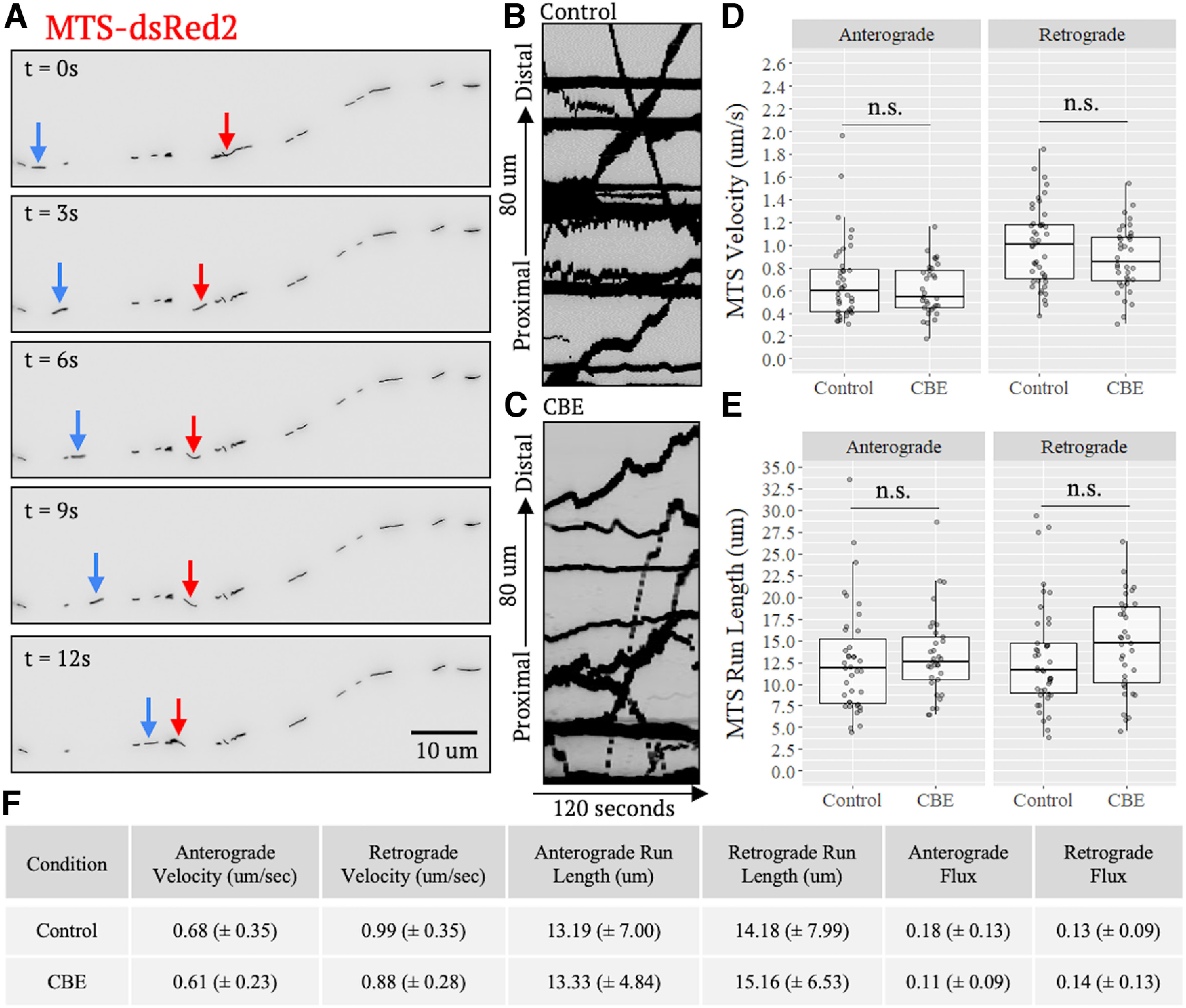
CBE does not affect axonal transport of mitochondria. 54 DIV neurons were transfected with mitochondrial marker MTS-dsRed2 and imaged 24 h later. ***A*** and Movie 2, Still frames of a representative axon from a control condition video at various time points and (***B***, ***C***) representative kymographs from one control and one CBE-treated MTS-dsRed2 video. Red arrows denote retrograde; blue arrows denote anterograde. Videos were acquired at 0.5 frames/s for 180 s. ***D***, ***E***, Analysis of kymographs revealed no significant effect of CBE treatment on any transport parameter (*N* = 3 independent experiments run in triplicate; Control, *n* = 40, 437 transport events; CBE, *n* = 35, 470 transport events, n.s. = nonsignificant, *p* > 0.05). ***F***, Table of all measured transport parameters (±SD) and Extended Data Table 1-1.

Notably, the loss of GCase activity causes mitochondrial dysfunction, where mitochondrial respiration, morphology, and notably, mitochondrial-lysosomal contact sites, are all altered by CBE treatment (Kim et al., 2021). Similarly, *GBA* deletions and mutations also lead to defects in mitochondrial respiration and morphology in neurons (H. Li et al., 2019; Morén et al., 2019). Because alterations in mitochondrial respiration influence mitochondrial transport (Hollenbeck et al., 1985; Miller and Sheetz, 2004), we tested whether mitochondrial transport would be disrupted by CBE treatment. Neurons were treated for 10 d with CBE and the resulting 54 DIV neurons were transfected with MTS-dsRed2, a mitochondrial marker, and allowed to express this marker for 24 h before imaging. Mitochondrial trafficking videos were acquired, converted to kymographs, and analyzed (Fig. 5*A*,*B*; Extended Data Table 1-1; Movie 2). The velocity, run length, and flux of the mitochondria in control neurons were consistent with previous reports (Fig. 5*C–E*; Extended Data Table 1-1; Miller and Sheetz, 2004). Furthermore, in both anterograde and retrograde directions, no significant effect was seen in CBE-treated neurons compared with controls in any of the transport parameters, similar to what was observed for lysosomes. However, mitochondrial velocity and flux was slightly lower in CBE-treated neurons as compared with controls in the retrograde direction, although the effect did not reach significance. These results show that axonal transport dynamics of mitochondria are not affected by CBE treatment.

Movie 2. Live imaging of axons showing MTS-dsRed2 transport in control versus CBE-treated cells. 0.5 frames/s, 90-s recording; playback: 4× real time. 10.1523/ENEURO.0079-23.2023.video.2

### CBE does not influence lysosomal rupturing

**Figure 6.**
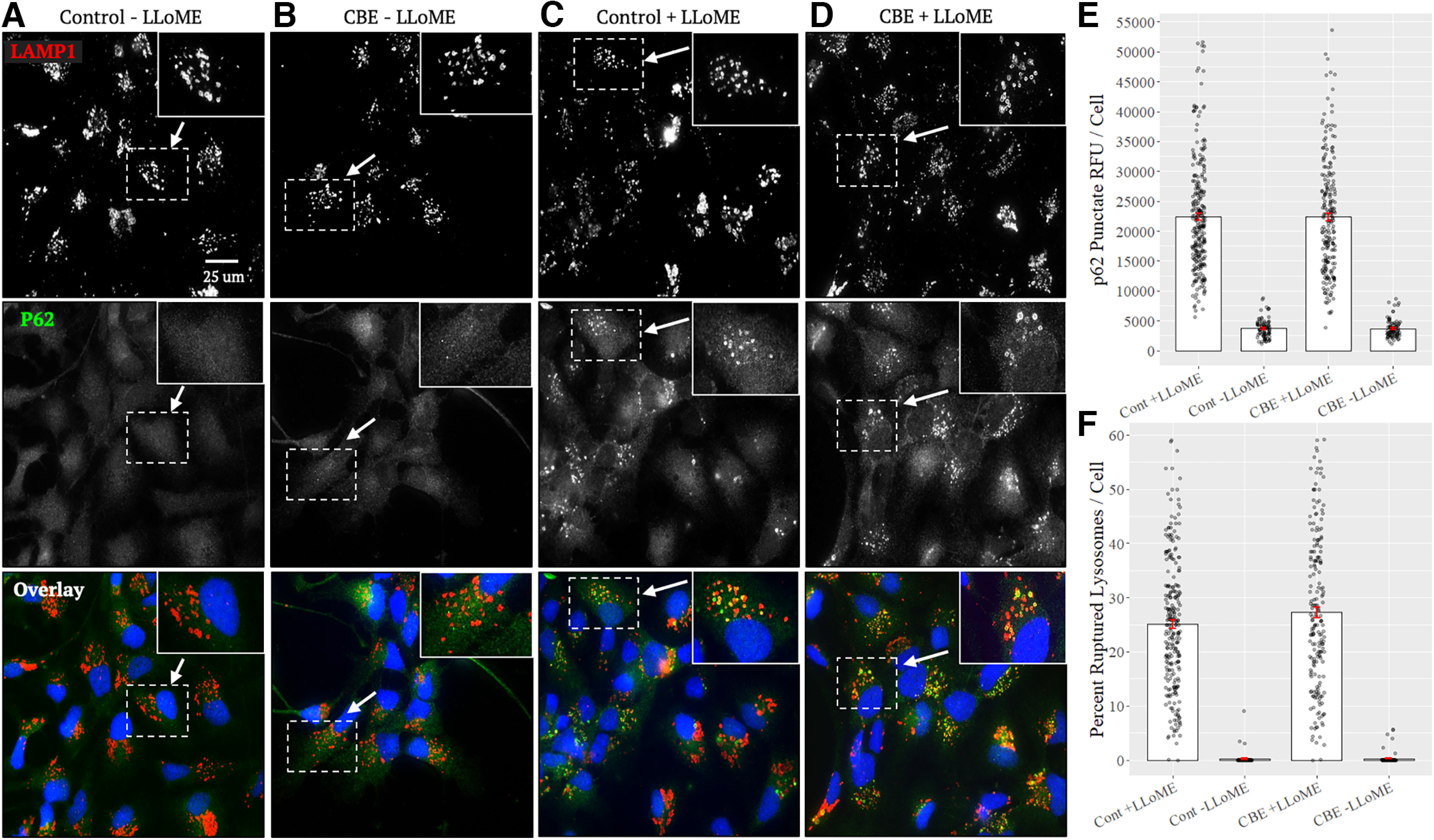
CBE does not influence lysosomal rupturing following LLoME treatment. CBE-treated NPCs and controls assessed for lysosomal rupture events by staining for LAMP1 (top, red) and p62 (middle, green), and colocalized (bottom). ***A***, ***B***, In the absence of LLoME, neither CBE nor control NPCs develop p62-positive lysosomes. ***C***, ***D***, Following treatment of 600 μm LLoME for 1 h, p62 puncta form in both CBE and control conditions. ***E***, p62-positive puncta colocalizing with LAMP1 puncta were analyzed using the ImageJ plugin *ComDet*, revealing no significant difference in puncta relative fluorescence units (RFU) per cell. ***F***, The percent of LAMP1 positive puncta that colocalized with p62 puncta, indicating lysosomal rupture, per cell were analyzed using *ComDet*. There was no significant difference in the percent ruptured lysosomes per cell between controls and CBE-treated NPCs (*N* = 3 independent experiments run in duplicate; Control +LLoME, *n* = 257 cells; Control −LLoME, *n* = 91 cells; CBE +LLoME, *n* = 201 cells; CBE −LLoME, *n* = 91 cells; Extended Data Table 1-1).

Lysosomal membrane rupture releases proteolytic enzymes causing nonspecific protein cleavage in the cytosol and disruption of proteostasis. The effect of GCase inhibition on lysosomal rupture has not been examined, but one study reported increased cytosolic activity of acid phosphatase, a lysosomal enzyme, in dopamine neurons of a GD mouse model, indicating increased lysosomal permeability and leakage (Soria et al., 2017). Additionally, lysosomal rupture is affected by changes in lysosomal membrane composition and lipid storage levels (Appelqvist et al., 2011; Liu et al., 2020). Thus, we hypothesized that inhibition of GCase would sensitize lysosomes to rupturing, which would offer mechanistic insight into how loss of GCase activity could promote pathogenesis in *GBA*-related disorders. To perform these studies, we used LLoME, a lysosomotropic molecule that, after endocytosis and transport into lysosomes, is cleaved by the pH-dependent enzyme cathepsin C, converting it into a detergent that selectively permeabilizes the lysosomal membrane. The resulting damaged lysosomes become coated by the autophagic cargo adaptor p62, which binds microtubule-associated protein 1A/1B light chain 3B (LC3) on autophagosomes, causing the autophagic engulfment of the lysosome and its degradation (lysophagy). Immunocytochemical labeling of p62 and LC3 is routinely used to selectively identify ruptured lysosomes (Otomo and Yoshimori, 2017; Liu et al., 2020). NPCs were used in this assay as when this was tested in neurons, no p62 and LC3 aggregates were seen (data not shown) possibly because of limited cathepsin C expression in neurons (Fan et al., 2012), a necessary prerequisite for cleavage and activation of LLoME. NPCs were treated with CBE for 10 d and a 1-h treatment of 600 μm LLoME was performed before fixing, staining them with p62, and LAMP1, and imaging; 600 μm LLoME was used, as previous studies conducted dose–response curves to show that at this dosage submaximal lysosomal and autophagic rupture is seen, with ∼1000 μm being a maximal dosage (Maejima et al., 2013; Eriksson et al., 2020). Images were analyzed using the ImageJ plugin *ComDet*, to determine the average intensity of p62 puncta and the percent of LAMP1 puncta that colocalized with p62 puncta. Although p62-positive LAMP1 vesicles were observed in cells from all LLoME-treatment conditions, we found no significant difference in the intensity of p62 puncta or the percentage of LAMP1 puncta that colocalize with p62 puncta between CBE-treated NPCs and controls (Fig. 6*A–F*; Extended Data Table 1-1). As confirmation, we performed another experiment using LC3 staining intensity as an indicator of lysosomal rupture, but similarly did not observe any change in the intensity of LC3 puncta or percent of LAMP1 puncta colocalizing with LC3 between conditions (Extended Data Fig. 1-1; Extended Data Table 1-1). These results suggest that CBE treatment does not alter the propensity of lysosomes to rupture following application of LLoME.

## Discussion

Though major efforts have been made and various proposals advanced, the mechanisms by which GCase dysfunction promotes neurodegeneration remain ill-defined. Here, through direct assessment of organelle trafficking at high spatial and temporal resolution in iPSC-derived forebrain neurons, we demonstrate that CBE has no significant effect on axonal lysosomal transport dynamics (Figs. 3, 4). A slight, but not statistically significant, decrease in retrograde transport dynamics was noted in CBE-treated neurons (Figs. 3, 4). Additionally, mitochondrial-specific processes, such as respiration, are affected by CBE-treatment which we reasoned may in turn affect transport dynamics. We found, however, no significant difference in transport dynamics of mitochondria in CBE-treated neurons (Fig. 5). Finally, rupture of lysosomes is another process affected by changes in lysosomal membrane composition. As such, we used an *in vitro* biochemical LLoME assay to assess changes in susceptibility to rupture in CBE-treated NPCs. Again, we observed no significant changes between conditions (Fig. 6). From these findings, we conclude that GCase inhibition via CBE-treatment likely does not affect the various lysosomal processes of transport and rupture.

Reports on the effects of GCase deficiency on lysosomal processes are at times contradictory (Dermentzaki et al., 2013; Bae et al., 2015). These discrepancies likely arise because of the various methods in which GCase deficiency is modeled in a variety of different systems, including using various *GBA* mutations, *GBA* knock-outs, and pharmacological inhibition of GCase via CBE in varying cell types and animal models (Taguchi et al., 2017; Henderson et al., 2020). Furthermore, pharmacological inhibition of CBE, as used in this study, has been applied at different dosages and durations leading to varying results when assessing lysosomal function. Here, we used a conservative dosage and duration (100 μm for 10 d), which is sufficient for GCase inhibition and GlcCer accumulation in neuronal cultures (Korkotian et al., 1999; Henderson et al., 2020). Notably, higher doses of CBE causes inhibition of additional enzymes including *GBA*2, acid α-glucosidase, β-glucuronidase, and consequently causes various off-target effects (Kuo et al., 2019). Given these dynamics, we predict that longer CBE treatment regiments (∼30–40 d) could sufficiently alter membrane composition to impinge on transport dynamics, but the physiological relevance of such a treatment is questionable.

Although we found no significant differences in lysosomal and mitochondrial transport between conditions, interestingly, LAMP1-GFP labeled vesicles did show a slight reduction in retrograde transport when compared with LysoBrite labeled vesicles (Figs. 3, 4). This suggests that transport deficiencies may be limited to predegradative subpopulations of LAMP1 positive structures, such as late endosomes or late autophagosomes. Mechanistically, this could be a consequence of GlcCer accumulation as, in GD cell lines, lysosomal GlcCer spills over into endosomal compartments (Hein et al., 2007). Further, the effect of GlcCer on vesicle membrane fluidity, a biophysical property known to influence transport efficacy (Grover et al., 2016), is dependent on vesicle pH. GlcCer promotes membrane rigidity at neutral pH, but membrane fluidity and tubulation at acidic pH (Varela et al., 2016). These pH-dependent properties of GlcCer could interfere with motor protein attachment or regulation on preacidic endosomes, where GlcCer levels are normally low, but not mature acidic lysosomes.

Lysosomal rupturing is another important pathogenic process in models of synucleinopathies that depends on lysosomal membrane composition (Appelqvist et al., 2011; Liu et al., 2020). However, we saw no significant change in lysosomal rupture between conditions in our model (Fig. 6). The prion-like spread of α-synuclein, a key process in disease progression of synucleinopathies, is dependent on lysosomal rupture in certain models of disease (Jiang et al., 2017). Cells collect and sequester α-synuclein oligomers into lysosomes where the acidic environment stabilizes their oligomeric conformation (Stephens et al., 2019). Subsequently, oligomers induce the permeabilization and lysis of lysosomal membranes, enabling their escape and prion-like spread between neurons (Karpowicz et al., 2017). It is possible that within our model, no change in permeability was observed between conditions because GCase has a noncatalytic role in disease. For example, *GBA* interacts with α-synuclein as a folding chaperone independently of catalytic activity (Yap et al., 2011), and additional pathogenic factors, such as the presence of α-synuclein oligomers or aggregates, are likely required for acute lysosomal dysfunction in neurons. Supporting this notion, CBE treatment of cultured neurons enhances the aggregation of exogenous α-synuclein oligomers, but does not induce the aggregation of endogenous α-synuclein (Henderson et al., 2020). GCase deficiency may therefore indirectly exacerbate lysosomal dysfunctions like increased permeability or transport efficiency, but only as a secondary consequence of an existing pathology such as α-synuclein aggregation.

GCase deficiency stimulated by CBE-treatment did not result in any significant change in any of the lysosomal processes assayed. Although the absence of lysosomal dysfunction following CBE treatment is both supported and refuted in the literature, it is possible that GCase deficiency alone is not sufficient to recreate disease conditions in certain cellular and animal models. Future studies on GCase deficiency should investigate whether lysosomal parameters are affected in more physiologically representative models of disease, such as neuronal models that incorporate key proxies of synucleinopathies (e.g., α-synuclein oligomers, SNCA mutations, or microglial inflammation). Additionally, experimental conditions could be adjusted to more accurately represent disease parameters such as neuronal-microglial co-cultures, the use of purified populations of dopaminergic neurons, or iPSC cell lines with deletions or disease-causing mutations in *GBA*.

## Synthesis

Reviewing Editor: Eran Perlson, Tel Aviv University Decisions are customarily a result of the Reviewing Editor and the peer reviewers coming together and discussing their recommendations until a consensus is reached. When revisions are invited, a fact-based synthesis statement explaining their decision and outlining what is needed to prepare a revision will be listed below. The following reviewer(s) agreed to reveal their identity: Laura Volpicelli-Daley.

In this study the authors examine how axonal organelle transport is altered in stem-cell derived neurons upon inhibiting glucocerebrosidase using a pharmacological agent, Conduritol Epoxide (CBE). GCase deficiency causes Gaucher’s disease and mutations in gene encoding the enzyme have been linked to Parkinson’s disease. Given this, the research presented is of interest to the neuroscience and neurodegeneration field

The authors carry out assays to first establish the neuronal differentiation, then examine the dynamics and transport properties of endo-lysosomes using a couple of different probes in neurons where GCase activity has been inhibited. They also examine how mitochondrial dynamics in these neurons and the lysosomal membrane permeability using LLoMe as a sensitizing agent.

The study suggests that inhibition of GCase does not alter the axonal transport of LAMP1-GFP or MTS-dsRed/mitochondria in differentiated iPSCs. This is a potentially important study because the mechanisms by which reduced GCase activity in Gaucher’s disease or Parkinson’s disease cause phenotypes are unknown. Reductions in axonal transport are one likely mechanism and the data from this study seem to indicate that this is not the case.

Still, a few concerns need to be addressed prior to publication:

1. How were axons distinguished from dendrites? The kinetics of transport are very different in these organelles.

2. A major concern is with regard to the statistics and experimental repeats performed. Several experiments are described as data collected from 3 coverslips. It is unclear if these are technical replicates or biological replicates. Experiments from multiple independent differentiation should be performed to account for experimental variability. Even if the overall N is 3 for 3 independent repeats of the experiment. The t-test should have fields within experimental repeats. In other words, the overall N is not 120 (3 repeats, 40 fields per experiment). Likewise, some assays have N=2 replicates. Even if the data suggests there is no significant difference, at least 3 experimental repeats need to be performed. Lastly, while T-test is suitable for comparing two groups, in some cases more than 2 groups are compared (for instance Figure 6E, F and extended data Figure 1-1). Here, another statistical test such as ANOVA may be more appropriate. Please re-do all the analyses with the appropriate statistical method.

3. Please submit videos of LAMP1-GFP and MTS-dred for both control and CBE treatment. Please include video snapshots and kymographs of both the Control and CBE treated. In figures 4, 5 it is unclear what exactly the stills of kymogrpahs are showing, based on the description. Is this from one movie? If so, is it just a control condition? If so, please include similar still for the GCase-inhibited condition for each figure and clarify exact nature (one axon from XX condition starting at distal end etc).

4. For figure 6, 600 μM LLoME is a lot; my guess is that lysosomal rupture is maximal with this concentration. So to determine if the cells are sensitized by CBE a dose-response would be necessary. Regardless, the CBE neurons seem to have more p62- if so, this would be interesting and important. Is the method of quantitation reflecting what is actually occurring? Can the authors use fluorescent-tagged galectin-3 as another method for assaying lysosomal rupture?

5. I am not convinced that the LAMP1-GFP in axons is necessarily lysosomes and not late endosomes. The organelles do not have active proteases until they are closer to the soma. (see https://pubmed.ncbi.nlm.nih.gov/21613495/) . The pH-sensitive dyes have not convinced me that they are low pH lysosomes vs. slightly high pH endosomes. Regardless, I would like the authors to tone down their claims that these are lysosomes with active proteases. The more distal lamp1-gfp puncta are likely late endosomes.

6. While the staining for MAP2 and Tau in Figure 1 are quite convincing, the staining for synaptic proteins seems a little unconvincing -or suggestive of slightly immature neurons. This raises a concern as to whether the neurons are old or mature enough and if older/more mature neurons might be more sensitive to GCase inhibition. Given Parkinson’s disease is an age-related neurodegenerative disease, examining ‘older’ or at least mature neurons will strengthen the paper.

7. While the authors make a case for the specific concentration of inhibitor used, citing more nonspecific effects at higher concentrations, given they are providing evidence that contradicts studies in other models, testing other dosage or length of treatment-for both GCase specific inhibition as well as axonal lysosome transport is necessary prior to making a strong statement as in the title (that GCase inhibition doesn’t affect lysosome transport in these neurons).

## Author Response

Dear Dr. Perlson,

Thank you very much for the reviews of our manuscript entitled ““Axonal transport of lysosomes is unaffected in glucocerebrosidase-inhibited iPSC-derived forebrain neurons” by Keefe et al., (eN-NRS-0079-23). We are grateful that the reviewers have acknowledged the quality and potential impact of our work, and that you have conditionally accepted our manuscript pending revisions. Our detailed replies to specific comments (in italics) are provided below. Bold type represents textual changes introduced into the revised manuscript. Changes in the manuscript itself are denoted in red text.

Response to Reviewers’ Comments - Synthesis Statement for Author:

1. How were axons distinguished from dendrites? The kinetics of transport are very different in these organelles.

We apologize for missing this critical methodological point and have for many years made clear distinctions between the axon and dendrites using the following approach (Reviewer figure below; Gan and Silverman, 2015, Gan et al., 2020): Principally, the axon and dendrites were identified by morphological criteria during video acquisition, where the axon is characterized by its even caliber and long length compared to dendrites with their characteristic taper, tendency to branch closer to the cell body, and shorter overall length. Moreover, after each imaging session, for a sample of cells, we performed retrospective immunocytochemistry confirming process identity using Tau as an axonal marker and MAP2 as a dendritic marker. Thus, processes that were either Tau-positive or MAP2-negative were scored as an axon. Due to the challenge of identifying every live-imaged cell, e.g., LAMP1-GFP fluorescence can be reduced after fixation, finding the same cells again on the fixed coverslip, we assess every video and if there was any doubt of axon identity, that video was removed from the analysis; around 20%. Furthermore, during trafficking analysis of LAMP1-GFP and LysoBrite, we observed an enrichment of LAMP1-GFP positive vesicles in presumed dendrites and the axon initial segment versus a relative scarcity in distal axons. The increased density of LAMP1 and acidic vesicles in dendrites compared to axons is consistent with other literature (Boecker et al., 2020). We used these differences in vesicle density in addition to morphological characteristics to distinguish between axonal and dendritic compartments. Finally, videos were acquired approximately 150-200 m from cell bodies, a distance that largely excluded dendrites due to their shorter length.

We have included the following description in the Methods and Materials in lines 127-133; Axons were initially identified based on morphology and imaged approximately 150-200 m from the cell body. Subsequently, a sample of cells were immunostained after live imaging to retrospectively confirm axon identity either by colocalization with Tau (1:500, ThermoFisher, AHB0042), an axonal protein, or exclusion of MAP2 (1:700, Sigma, AB5622), a dendritic cytoskeletal protein. In order to determine the orientation of the cell body relative to the axon, and thus to distinguish between anterograde and retrograde transport events, only visually isolated axons were imaged.

2. A major concern is with regard to the statistics and experimental repeats performed. Several experiments are described as data collected from 3 coverslips. It is unclear if these are technical replicates or biological replicates. Experiments from multiple independent differentiation should be performed to account for experimental variability. Even if the overall N is 3 for 3 independent repeats of the experiment. The t-test should have fields within experimental repeats. In other words, the overall N is not 120 (3 repeats, 40 fields per experiment). Likewise, some assays have N=2 replicates. Even if the data suggests there is no significant difference, at least 3 experimental repeats need to be performed. Lastly, while T-test is suitable for comparing two groups, in some cases more than 2 groups are compared (for instance Figure 6E, F and extended data Figure 1-1). Here, another statistical test such as ANOVA may be more appropriate. Please re-do all the analyses with the appropriate statistical method.

We apologize for our statistical descriptions as we recognize they were inadequate. To address reviewer concerns regarding how we conducted and describe statistical analysis and experimental repeats, we have updated figure descriptions for Figures 2, 3, 4, 5, 6 and Extended Data Figure 1-1. In these cases, we define “N” as the number of independent experiments (biological replicates) with an attached description of technical replicates (duplicate/triplicate), and “n” as our sample size per condition. Furthermore, each independent experiment was conducted by thawing and differentiating a new sample of NPCs into mature, forebrain neurons as newly described in lines 104-105 “For each independent experiment, a new vial of NPCs was thawed and used for differentiation into terminally differentiated neurons.”

Furthermore, we agree with the reviewers regarding Figure 2E, in which we previously only conducted two independent experiments to highlight GFAP intensity differences between control and CBE conditions. As such, we have run a third independent experiment, and have updated Figure 2E and the corresponding figure description, as well as updated Table 1-1 to adequately describe experimental results and analysis parameters.

We have also addressed reviewers’ concerns regarding the type of statistical analyses run for Figure 6E, F and Extended Data Figure 1-1E, F. Lines 206 - 208 describe that “For lysosomal rupture experiments, we performed one-way ANOVAs with Tukey’s post hoc tests, comparing control and CBE-treated conditions within the same experiments.” We have included these statistics in Figure 6E, F and Extended Data Figure 1-1E, F, as well as Extended Data Table 1-1.

In summary, we have re-analyzed all data sets and generated new statistical values (sample sizes, averages, standard deviations, degrees of freedom, p-values) as recommended by the reviewers, which can be found in the figure descriptions for Figures 2-6 and Extended Data Figures 1-1 and Table 1-1.

3. Please submit videos of LAMP1-GFP and MTS-dsred for both control and CBE treatment. Please include video snapshots and kymographs of both the Control and CBE treated. In figures 4, 5 it is unclear what exactly the stills of kymogrpahs are showing, based on the description. Is this from one movie? If so, is it just a control condition? If so, please include similar still for the GCase-inhibited condition for each figure and clarify exact nature (one axon from XX condition starting at distal end etc).

As requested by the reviewers, we have submitted videos of both LAMP1-eGFP and MTS-dsRed for both Control and CBE-treated iPSC-derived forebrain neurons, for example in line 247 - (Fig 3A-B; Table 1-1; Movie 1). We have further included a sample kymograph for both the Control and CBE-treated conditions in Figures 3B, 4B and 5B, and have clearly labelled the directionality of the kymographs and stills and amended the descriptions to better explain what is shown.

4. For figure 6, 600 μM LLoME is a lot; my guess is that lysosomal rupture is maximal with this concentration. So to determine if the cells are sensitized by CBE a dose-response would be necessary. Regardless, the CBE neurons seem to have more p62- if so, this would be interesting and important. Is the method of quantitation reflecting what is actually occurring? Can the authors use fluorescent-tagged galectin-3 as another method for assaying lysosomal rupture?

We understand the reviewers concerns that 600 μM LLoME would result in maximal rupture. However, dose-response curves have been performed in a variety of cell lines previously and show that 600 μM LLoME is a sub-maximal dosage, with maximal lysosomal rupture being seen at 1000 μM LLoME. This dosage (1000 μM) is typically used in studies which require maximal lysosomal rupture to conduct their experiments. We have added a statement and references regarding the sub-maximal dose in line 304 - 307 stating “600 μM LLoME was used, as previous studies conducted dose-response curves to show that at this dosage sub-maximal lysosomal and autophagic rupture is seen, with ∼1000 μM being a maximal dosage (Maejima et al., 2013; Eriksson et al., 2020).”.

Regarding CBE-treated NPCs seemingly having more p62 punctate staining, we re-analyzed this data set per cell as well as using a one-way ANOVA with a post-hoc Tukey test to see whether there were any significant differences in the means between Control -LLoME and CBE -LLoME, as well as Control +LLoME and CBE +LLoME. Previously, we analyzed our data per field of view, which as the reviewers pointed out, may inadequately assess the effect CBE has on sensitizing NPCs to rupture. We also previously used an inadequate and incorrect statistical test, the two-tailed unpaired Student t-test, to look for differences between these groups. Using the correct test, we found that the differences in the staining were insignificant (Figure 6E: p=0.9999 and p=0.9952, respectively; Figure 6F: p=0.9999 and p=0.1492, respectively). We have amended the Materials and Methods section to reflect this change from analyzing the field of view to analyzing individual cells stating in lines 188-189 “Cells were outlined by hand using the ImageJ “Region of Interest (ROI)” tool and the subsequent ROIs were analyzed using the ImageJ plugin ComDet.”

Although we do recognize that fluorescently-tagged galectin-3 would be another way to assay lysosomal rupture, in Figure 1-1 we stained the NPCs for LC3, another commonly used marker of lysosomal rupture to confirm changes in lysosomal rupture that may have been missed with p62 staining. We chose this marker over galectin-3 as LC3 has been extensively used to assay pathophysiological phenomenon in GCase-deficiency related disorders such as Gaucher’s Disease (Mazzulli et al., 2011; de la Mata et al., 2015; Aflaki et al., 2016; Brown et al., 2019; Zhao et al., 2021). Similar to p62, comparing both Control -LLoME and CBE -LLoME, as well as Control +LLoME and CBE +LLoME we found no significant differences in rupture (Figure 1-1E: p=0.9902 and p=0.2365, respectively; Figure 1-1F: p=0.9371 and p=0.9274, respectively). We have amended the Materials and Methods section to reflect this change from analyzing the field of view to analyzing individual cells stating in lines 188-189 “Cells were outlined by hand using the ImageJ “Region of Interest (ROI)” tool and then analyzed using the ImageJ plugin ComDet.”.

5. I am not convinced that the LAMP1-GFP in axons is necessarily lysosomes and not late endosomes. The organelles do not have active proteases until they are closer to the soma. (see https://pubmed.ncbi.nlm.nih.gov/21613495/) . The pH-sensitive dyes have not convinced me that they are low pH lysosomes vs. slightly high pH endosomes. Regardless, I would like the authors to tone down their claims that these are lysosomes with active proteases. The more distal lamp1-gfp puncta are likely late endosomes.

We agree that axonal vesicles containing LAMP1-GFP are not necessarily lysosomes, and likely represent a broader population of endocytic vesicles of varying maturity. We used the pH-sensitive probe LysoBrite to complement our LAMP1-GFP transport assays but, as the reviewer correctly mentions, these too are not specific to lysosomes. Although pH-sensitive probes are a better marker for lysosomes than LAMP-GFP, they also label slightly acidic endosomes and autophagosomes. However, regardless of vesicle identity, and as the reviewers understand, their axonal transport is biologically significant and relevant to disease pathogenesis. We have amended our introduction to clearly communicate the above-mentioned ambiguities in vesicle identity implied with our usage of the term lysosome, and we instead emphasize to readers the biological importance and disease relevance associated with the transport of these vesicles. This can be seen in lines 39-41 “In the axon, lysosomes and other organelles such as late endosomes or autophagosomes (collectively referred to as lysosomes for simplicity), survey synapses in search of damaged material, which they collect and prepare for degradation.” and lines 243-244 “...and pre-degradative lysosomal populations (late endosomes and autophagosomes)”.

Other sentences have been amended which we hope will either clarify or soften the language when referring to lysosome-related populations. Line 254 now reads: Overall, about 20% of LAMP1 vesicles within the axon colocalize with LysoTracker, a marker that primarily stains mature lysosomes (Gowrishankar et al., 2021).

And in lines 261-263 the following has been added: Of note, although LysoBrite and other pH sensitive dyes are more specific for lysosomes than LAMP1-GFP, they also may label slightly acidic autophagosomes or late endosomes.

6. While the staining for MAP2 and Tau in Figure 1 are quite convincing, the staining for synaptic proteins seems a little unconvincing -or suggestive of slightly immature neurons. This raises a concern as to whether the neurons are old or mature enough and if older/more mature neurons might be more sensitive to GCase inhibition. Given Parkinson’s disease is an age-related neurodegenerative disease, examining ‘older’ or at least mature neurons will strengthen the paper.

We agree with the reviewers regarding the notion that age-related disorders such as Parkinson’s disease and Dementia with Lewy Bodies would better be served with more mature neuron’s, typically between 6-12 weeks post-onset of maturation (Lam et al., 2017; Gunhanlar et al., 2018; Togo et al., 2021). However, we chose the time point of 30 days post onset of differentiation for three reasons. Primarily, experiments probing GCase-deficiency related disorders such as Gaucher’s disease have previously used iPSC-derived neurons as early as two weeks post-maturation induction (Srikanth et al., 2021; Messelodi et al., 2023). Accordingly, morphological, biochemical, and functional polarization is achieved, i.e., differential distribution of dendritic and axonal proteins and action potential firing, in as early as two weeks post-maturation induction (Lam et al., 2017; Bell et al., 2019, Lindhout et al., 2020). Furthermore, functional staining of synapsin between similar and more mature time points is quite variable between culture protocols in the literature (Lam et al., 2017; Gunhanlar et al., 2018). Hence, we deferred to the polarization of key cytoskeletal proteins, i.e., MAP2, Tau, and the presence of synapsin as a marker of terminal differentiation as defined in the culture protocol of the STEMDiff Forebrain Maturation Kit (STEMCELL Technologies, Catalog #08605). Secondly, Gaucher’s disease, the most prevalent disease caused by mutations in the GBA gene, typically shows an early-onset, with the most commonly occurring Type 1 GD presenting in 50% of patients before the age of 10, and Type 2 presenting before the age of two (Kallish and Kaplan, 2013; Nagral, 2014). Lastly, although mitotic inhibitors were present in our differentiation and maturation protocols, we wanted to minimize the possibility of glial overgrowth in our culture, as glial overgrowth would limit neuronal interrogation due to the high spatiotemporal resolution needed for live imaging of lysosomal movement events and puncta quantification. As such, we believe that four weeks post-maturation induction of forebrain neurons was adequate to probe for potential physiologically relevant pathologic perturbations of lysosomal function.

7. While the authors make a case for the specific concentration of inhibitor used, citing more nonspecific effects at higher concentrations, given they are providing evidence that contradicts studies in other models, testing other dosage or length of treatment-for both GCase specific inhibition as well as axonal lysosome transport is necessary prior to making a strong statement as in the title (that GCase inhibition doesn’t affect lysosome transport in these neurons).

As mentioned, we refrained from using higher CBE doses due to possible nonspecific effects of CBE. We believe a higher dose was not warranted because we found 100 uM was sufficient to significantly reduce GCase activity relative to controls corroborating other studies using the same concentration for GCase inhibition (Henderson et al., 2020). Moreover, we used live cell imaging of lysosomal GCase activity using LysoFQ-GBA and this showed a significant decrease, consistent with blockade of GCase by CBE (Figure 2). Longer durations of CBE treatment were considered but decided against for the following reasons. Because our cultures required a 30-day maturation period, we were concerned that longer durations of CBE beginning at earlier time points of maturation could interfere with neuronal development. Additionally, we used a duration of CBE treatment that was similar to other studies, and in these other studies this dose was sufficient to elicit cellular dysfunction. Although we did not observe dysfunction in our measured variables, i.e., transport or rupture, the increase in astrocyte GFAP immunostaining demonstrated the bioactivity of CBE at the dose and duration used. Although longer durations could have been used, we did not consider it practical or necessary for this study to repeat all transport experiments at a higher dose which was not obvious to ascertain. Regardless, we did assess the effect of longer durations of CBE up to 25 days on neurons and found no apparent differences in protein aggregation (indicated by LC3 and p62 immunocytochemistry) compared to neurons treated with CBE for 10 days. These experiments suggest extended durations of CBE do not prompt novel cellular pathology, and therefore we did not anticipate longer CBE treatment durations would differentially affect transport or rupture.

We appreciate the constructive critiques of the reviewers and feel that these comments have substantially improved our work. We now hope the current version of our manuscript is considered acceptable for publication in eNeuro.

-

References

Aflaki E, Moaven N, Borger DK, Lopez G, Westbroek W, Chae JJ, Marugan J, Patnaik S, Maniwang E, Gonzalez AN, Sidransky E (2016) Lysosomal storage and impaired autophagy lead to inflammasome activation in Gaucher macrophages. Aging Cell 15:77-88.

Bell S, Hettige NC, Silveira H, Peng H, Wu H, Jefri M, Antonyan L, Zhang Y, Zhang X, Ernst C (2019) Differentiation of Human Induced Pluripotent Stem Cells (iPSCs) into an Effective Model of Forebrain Neural Progenitor Cells and Mature Neurons. Bio Protoc 9:e3188.

Boecker CA, Olenick MA, Gallagher ER, Ward ME, Holzbaur ELF (2020) ToolBox: Live Imaging of intracellular organelle transport in induced pluripotent stem cell-derived neurons. Traffic 21:138-155.

Brown RA, Voit A, Srikanth MP, Thayer JA, Kingsbury TJ, Jacobson MA, Lipinski MM, Feldman RA, Awad O (2019) mTOR hyperactivity mediates lysosomal dysfunction in Gaucher’s disease iPSC-neuronal cells. Dis Model Mech 12:dmm038596.

de la Mata M, Cotán D, Oropesa-Ávila M, Garrido-Maraver J, Cordero MD, Villanueva Paz M, Delgado Pavón A, Alcocer-Gómez E, de Lavera I, Ybot-González P, Paula Zaderenko A, Ortiz Mellet C, García Fernández JM, Sánchez-Alcázar JA (2015) Pharmacological Chaperones and Coenzyme Q10 Treatment Improves Mutant β-Glucocerebrosidase Activity and Mitochondrial Function in Neuronopathic Forms of Gaucher Disease. Sci Rep 5:10903.

Gan KJ and Silverman MA. Dendritic and axonal mechanisms of Ca2+ elevation impair BDNF transport in A→ oligomer-treated hippocampal neurons. Mol Biol Cell, 2015 Mar 15;26(6):1058-71.

Gan, KJ, Akram, A, Blasius, TL, Ramser, EM, Budaitis, BG, Gabrych, DR, Verhey, KJ, Silverman, MA. GSK3ß impairs KIF1A transport in a cellular model of Alzheimer’s disease but does not regulate motor motility at S402. eNeuro. 2020 Nov 5;7(6)

Gunhanlar N, Shpak G, van der Kroeg M, Gouty-Colomer LA, Munshi ST, Lendemeijer B, Ghazvini M, Dupont C, Hoogendijk WJG, Gribnau J, de Vrij FMS, Kushner SA (2018) A simplified protocol for differentiation of electrophysiologically mature neuronal networks from human induced pluripotent stem cells. Mol Psychiatry 23:1336-1344.

Henderson MX, Sedor S, McGeary I, Cornblath EJ, Peng C, Riddle DM, Li HL, Zhang B, Brown HJ, Olufemi MF, Bassett DS, Trojanowski JQ, Lee VMY (2020) Glucocerebrosidase activity modulates neuronal susceptibility to pathological α-synuclein insult. Neuron 105:822-836.e7.

Kallish S, Kaplan P (2013) A disease severity scoring system for children with type 1 Gaucher disease. Eur J Pediatr 172:39-43.

Lam RS, Töpfer FM, Wood PG, Busskamp V, Bamberg E (2017) Functional Maturation of Human Stem Cell-Derived Neurons in Long-Term Cultures. PLoS One 12:e0169506.

Lindhout FW, Kooistra R, Portegies S, Herstel LJ, Stucchi R, Snoek BL, Altelaar AM, MacGillavry HD, Wierenga CJ, Hoogenraad CC (2020) Quantitative mapping of transcriptome and proteome dynamics during polarization of human iPSC-derived neurons. Elife 9:e58124.

Messelodi D, Strocchi S, Bertuccio SN, Baden P, Indio V, Giorgi FM, Taddia A, Serravalle S, Valente S, di Fonzo A, Frattini E, Bernardoni R, Pession Annalisa, Grifoni D, Deleidi M, Astolfi A, Pession Andrea (2023) Neuronopathic Gaucher disease models reveal defects in cell growth promoted by Hippo pathway activation. Commun Biol 6:431.

Mazzulli JR, Xu Y-H, Sun Y, Knight AL, McLean PJ, Caldwell GA, Sidransky E, Grabowski GA, Krainc D (2011) Gaucher disease glucocerebrosidase and α-synuclein form a bidirectional pathogenic loop in synucleinopathies. Cell 146:37-52.

Nagral A (2014) Gaucher Disease. J Clin Exp Hepatol 4:37-50.

Srikanth MP, Jones JW, Kane M, Awad O, Park TS, Zambidis ET, Feldman RA (2021) Elevated glucosylsphingosine in Gaucher disease induced pluripotent stem cell neurons deregulates lysosomal compartment through mammalian target of rapamycin complex 1. Stem Cells Transl Med 10:1081-1094.

Togo K, Fukusumi H, Shofuda T, Ohnishi H, Yamazaki H, Hayashi MK, Kawasaki N, Takei N, Nakazawa T, Saito Y, Baba K, Hashimoto H, Sekino Y, Shirao T, Mochizuki H, Kanemura Y (2021) Postsynaptic structure formation of human iPS cell-derived neurons takes longer than presynaptic formation during neural differentiation in vitro. Mol Brain 14:149.

Zhao X, Liberti R, Jian J, Fu W, Hettinghouse A, Sun Y, Liu C-J (2021) Progranulin associates with Rab2 and is involved in autophagosome-lysosome fusion in Gaucher disease. J Mol Med (Berl) 99:1639-1654.
