# Supplementary figures and images for: Axonal Transport of Lysosomes Is Unaffected in Glucocerebrosidase-Inhibited iPSC-Derived Forebrain Neurons
Source: eNeuro. 2023 Oct 6;10(10):ENEURO.0079-23.2023. doi: 10.1523/ENEURO.0079-23.2023 (PMC10576257; doi:10.1523/ENEURO.0079-23.2023)

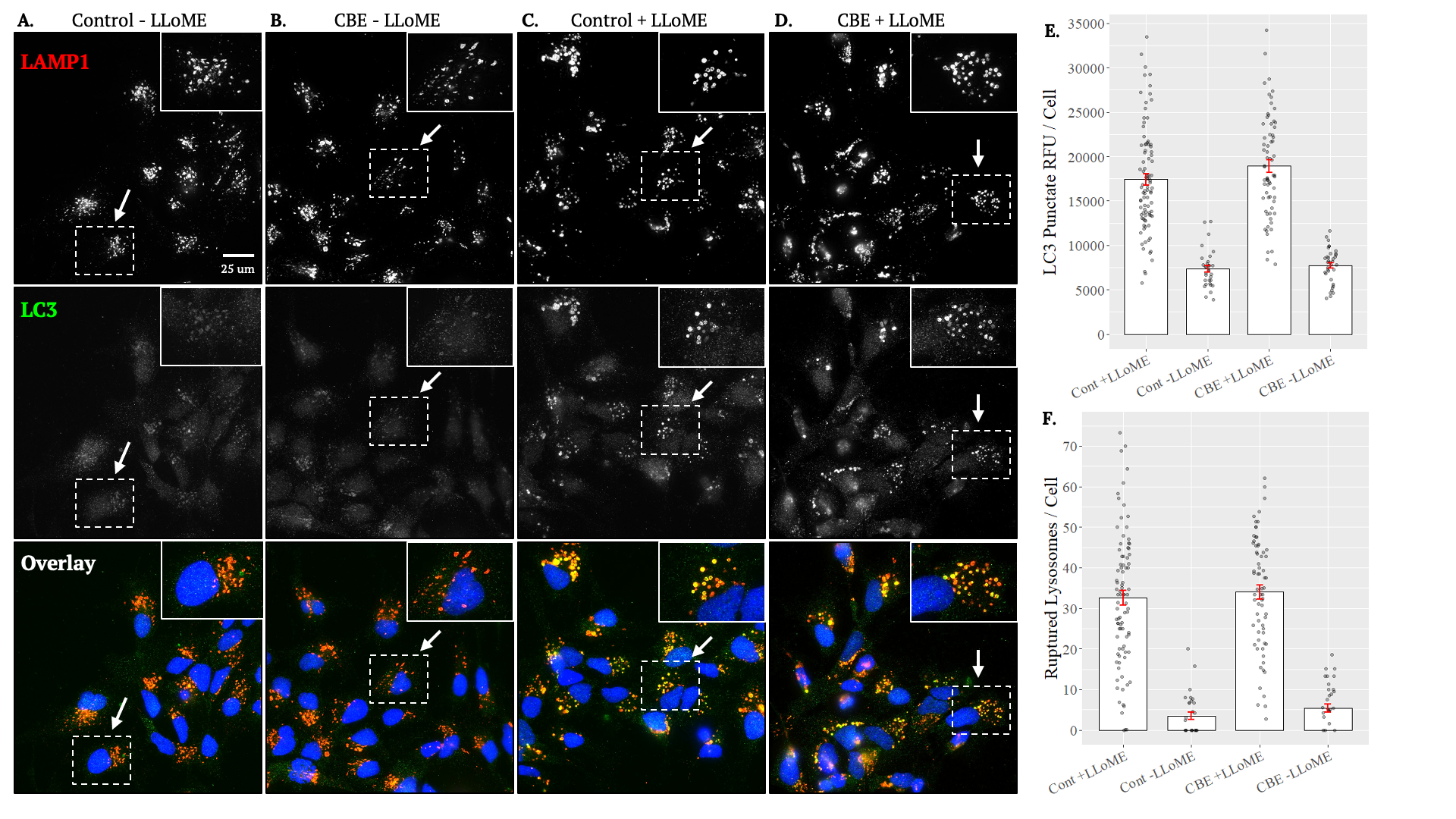

Supplement: Extended Data Figure 1-1 — CBE does not influence LC3 staining following LLoME treatment. CBE-treated NPCs and controls assessed for lysosomal rupture events by staining for LAMP1 (top, red) and LC3 (middle, green), and colocalized (bottom). A, B, In the absence of LLoME, neither CBE nor control NPCs develop LC3-positive lysosomes. C, D, Following treatment of 600 μm LLoME for 1 h, LC3 puncta form in both CBE and control conditions. E, LC3-positive puncta colocalizing with LAMP1 puncta were analyzed using the ImageJ plugin ComDet, revealing no significant difference in puncta relative fluorescence units (RFU) per cell. F, The percent of LAMP1 positive puncta that colocalized with LC3 puncta, indicating lysosomal rupture, per cell were analyzed using ComDet. There was no significant difference in the percent ruptured lysosomes per cell between controls and CBE-treated NPCs (N = 3 independent experiments run in duplicate; Control +LLoME, n = 90 cells; Control −LLoME, n = 32 cells; CBE +LLoME, n = 68 cells; CBE −LLoME, n = 36 cells; Extended Data Table 1-1). Download Figure 1-1, TIF file. [file enu-eN-NRS-0079-23-s02.tif]

Table 1-1


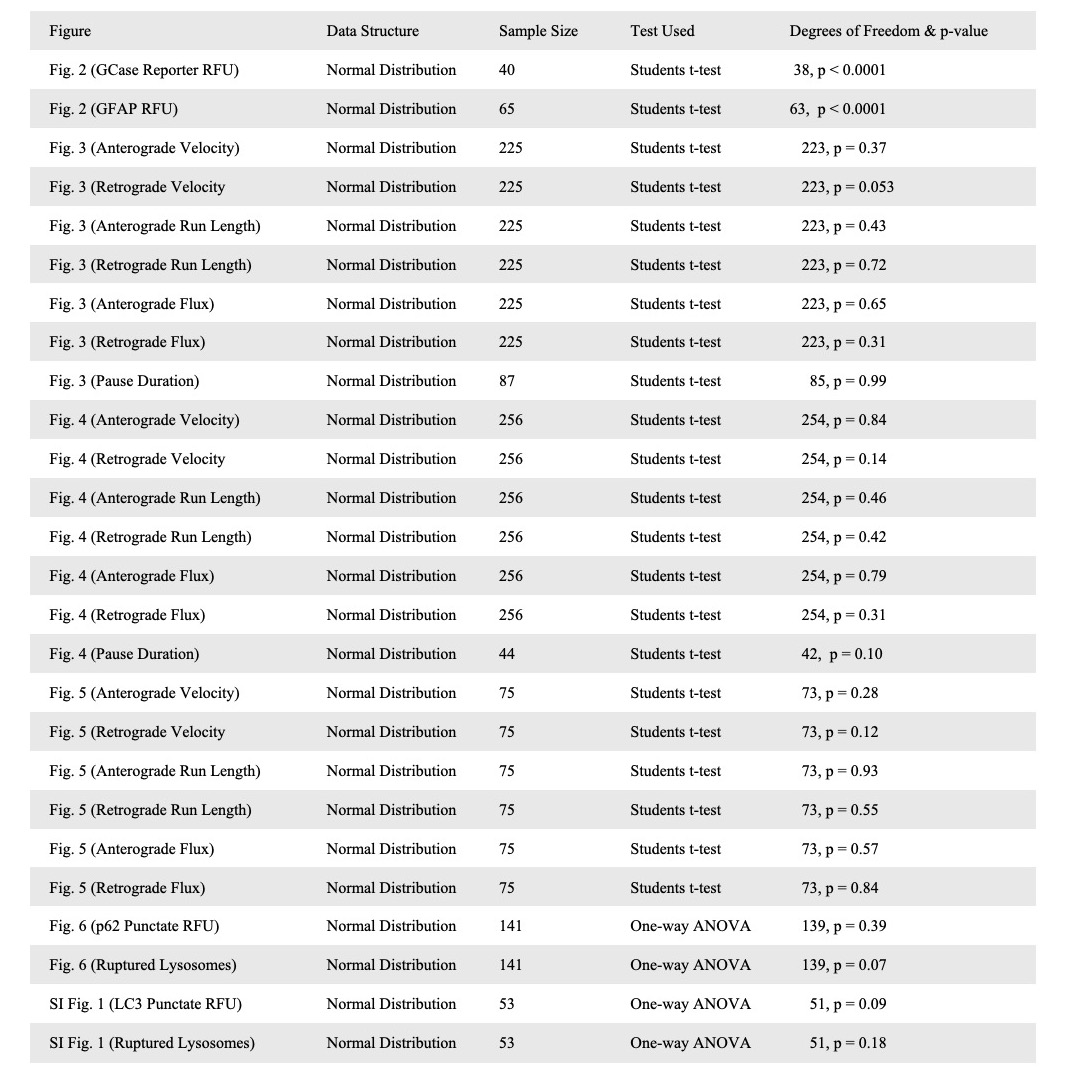

Supplement: Extended Data Table 1-1 — Student t test p values for all data. Download Table 1-1, DOC file. [file enu-eN-NRS-0079-23-s05.doc]
